# Strontium isotopic signatures and source apportionment in urban soils of Salgótarján: unraveling the impact of industrial emissions

**DOI:** 10.1007/s11356-026-38225-6

**Published:** 2026-09-23

**Authors:** Mona Maghsoudlou, Davaakhuu Tserendorj, Gorkhmaz Abbaszade, Norbert Kavasi, Péter Völgyesi, Edit Tóth-Bodrogi, Sarata K. Sahoo, Marko Štrok, Kazumasa Inoue, Tibor Kovács, Csaba Szabó

**Affiliations:** 1 Lithosphere Fluid Research Lab, Institute of Geography and Geology, Eötvös Loránd University, Pázmány Péter Sétány 1/C, Budapest, 1117 Hungary; 2 Geographical Institute, Research Centre for Astronomy and Earth Sciences, HUN-REN, Budaőrsi Út 45, Budapest, H-1112 Hungary; 3 Nuclear Security Department, Centre for Energy Research, HUN-REN, Konkoly Thege Út 29-33, Budapest, 1121 Hungary; 4 Flow Cytometry Group, Department of Applied Microbial Ecology, Helmholtz Centre for Environmental Research GmbH – UFZ, Permoserstraße 15, 04318 Leipzig, Germany; 5 Regional Environmental Co-Creation Unit, Fukushima Institute for Research, Education and Innovation, Fukushima, 960-1295 Japan; 6 Department of Environmental Sciences, Jožef Stefan Institute, 1000 Ljubljana, Slovenia; 7 Institute of Radiochemistry and Radioecology, University of Pannonia, Veszprém, Hungary; 8 Tokyo Metropolitan University, 7-2-10 Higashiogu, Arakawa-Ku, Tokyo, 116-8551 Japan; 9 National Institutes for Quantum Sciences and Technology (QST), 4-9-1 Anagawa, Inage-Ku, Chiba, 263-8555 Japan; 10 Institute of Earth Physics and Space Science, HUN-REN, Sopron, Hungary; 11 Centre of Environmental Sciences, Eötvös Loránd University, Pázmány Péter Sétány 1/A, 1117 Budapest, Hungary

**Keywords:** ^87^Sr/^86^Sr isotopic ratio, Urban soils, Coal-fired power plant (CFPP), Coal ash, Land use changes

## Abstract

Urban soil contamination is primarily associated with human activities, especially industrial by-products, which have introduced high concentrations of heavy metal(loid)s from diverse sources over centuries of city development. This study investigated the distribution of strontium and its stable isotopic ratios (^87^Sr/^86^Sr) in urban soils of Salgótarján, a former industrial city in northern Hungary, with a focus on anthropogenic contamination, particularly from a former coal-fired power plant (CFPP). Fifteen urban soil samples from kindergartens, playgrounds, parks, and roadsides, in the northeastern part of the city were analyzed for Sr concentration, isotopic composition, and physicochemical properties. The analytical results revealed considerable variations in Sr concentrations (17.5–51.3 mg kg⁻^1^), and ^87^Sr/^86^Sr ratios (0.710303–0.719559), indicating mixed natural and anthropogenic inputs. A two-end-member ^87^Sr/^86^Sr isotopic model was applied to semi-quantitatively estimate source contribution, identifying coal ash as a major contamination source in soils near the former CFPP. However, certain samples gathered far from the CFPP exhibited even stronger anthropogenic influence, suggesting an additional source, likely from smelter slag (known additional industrial source in the environment) and/or unknown technogenic soils (human-transformed soils). In contrast, other samples at greater distances from the CFPP showed minimal or no coal ash contribution, instead reflecting local soil inputs (i.e., natural/artificially made quartz-rich soils), introduced during land use changes. Our study highlights the effectiveness of ^87^Sr/^86^Sr isotopic tracing in urban contamination assessment, offering a robust tool for understanding and managing urban environment quality.

**Supplementary Information:**

The online version contains supplementary material available at https://doi.org/10.1007/s11356-026-38225-6.

## Introduction

Urban soil is produced as a result of urbanization, which has significant direct and indirect impact on environmental quality and human well-being (Craul 1985; Lehmann and Stahr 2007). Beyond serving as a physical substrate for infrastructure and human activities, urban soils play a crucial role in (bio)geochemical cycling of elements in the lithosphere (e.g., pedosphere), hydrosphere, atmosphere, as well as in fluid transport, growing plants, and recreational activities, etc. (Iqbal and Shah 2011; Luo et al. 2012). However, urban environments are significantly affected by complex anthropogenic inputs particularly those associated with historical industrial activities, such as mining, industrial metallurgical processes, coal combustion, and transportation (e.g., Kachenko and Singh 2006; Yang et al. 2009). Among these, coal-fired power plants (CFPPs) have long been recognized as major contributors to heavy metal(loid)s contamination in soils surrounding industrial areas (e.g., Finkelman 1999). The combustion of coal results in the accumulation of potentially toxic elements (PTEs) such as Pb, As, Cd, and Hg, which can persist in the environment for decades (e.g., Meij and Te Winkel 2004). These contaminants are primarily associated with coal ash, which deposits in surrounding soils, altering their chemical composition and potentially increasing ecological and human health risks (da Silva Júnior et al. 2019). The long-term impact of CFPP emissions is well-documented in industrial regions, where soil contamination patterns mirror historical operational practices and waste disposal strategies. This underscores the necessity of robust geochemical tracers to effectively distinguish between natural and anthropogenic sources (Ilić et al. 2022). Salgótarján, a major heavy industrial center which played significant economic role in Hungary by developing multiple industrial activities, began with brown coal mining in the mid-19th. The industrial legacy from coal mining followed and developed by significant and extensive (at least 170 years) heavy industrial activities (i.e., coal-fired power plant, metal smelting, glass works and mining machinery) (Horváth and Csüllög, 2012). After the collapse of the communist system, industrial structure evolved in the early 1990 s and coal mines and heavy industries declined. Large amount of industrial wastes, coal mine waste dumps, coal ash and smelter slag deposits remained throughout the city. The landscape of the city underwent considerable changes because of economic and social change, which included the emergence of the reconstruction of public facilities like playgrounds and parks, as well as commonwealth institutions, like kindergartens and schools. However, in several cases, the above-mentioned waste materials were repurposed as filling materials during urban development which, in turn, resulted in unfavorable landscape modifications and negative consequences for the entire city.

To assess and trace these complex anthropogenic pollution from above-mentioned industrial activities, various environmental indicators can be used, including elemental distribution, stable isotopic ratios, radioactivity levels, as well as different key physical and chemical features (e.g., grain size fractions, pH, and Eh values) of urban soils (e.g., Hagan et al. 2012; Turnbull et al. 2019; Malone et al. 2023). Among them, stable isotopes serve as particularly valuable and powerful tools for tracing contamination and assessing urban environmental conditions in the context of sustainable development, and monitoring ecosystem changes (e.g., Charalampides and Manoliadis 2002; Olowoyo et al. 2022; Tešić et al. 2022; Kelepertzis et al. 2023).

Strontium is a large ionic lithophile element, moderately incompatible in solid phases and considered mobile in fluid transport systems in the environment (e.g., Casey et al. 2018). Among its four naturally occurring stable isotopes (^88^Sr: 82.6%, ^87^Sr: 7.0%, ^86^Sr: 9.9%, ^84^Sr: 0.6%), the proportion of ^87^Sr is influenced by radiogenic processes (^87^Rb beta decay, half-life of 48.8 billion years), showing a continuous increase in the geogenic environment. Therefore, the ^87^Sr/^86^Sr ratio is typically elevated in the natural environment compared to anthropogenic by-products (such as coal ash) (e.g., Clauer et al. 2021). Furthermore, enrichment of Sr in coal ash indicates that soils mixed with coal ash exhibit higher concentration of Sr than natural soils (e.g., Cochran et al. 2018). Recent studies have increasingly used ^87^Sr/^86^Sr isotopic analysis as promising method for tracing coal ash contamination in urban environment (Hurst et al. 1991; Sherman et al. 2015). This study investigates ^87^Sr/^86^Sr ratios in urban soil samples from Salgótarján (a former industrial city in North Hungary) to identify the contamination sources and estimate the extent of anthropogenic influence from past industrial activities within and around residential areas.

The major goals are: 1) to determine the distribution of strontium concentrations in urban soils of Salgótarján and examine their correlations with some key physicochemical properties of soil, including pH, total organic carbon (TOC), total nitrogen (TN), phosphorus (P), sulfur (S), and grain size distribution; 2) to characterize variations in ^87^Sr/^86^Sr ratios as a function of distance from the local coal-fired power plant (CFPP); and 3) to estimate the relative contributions of major contamination source(s) in the study area.

## Material and methods

### Study area

**Fig. 1:**
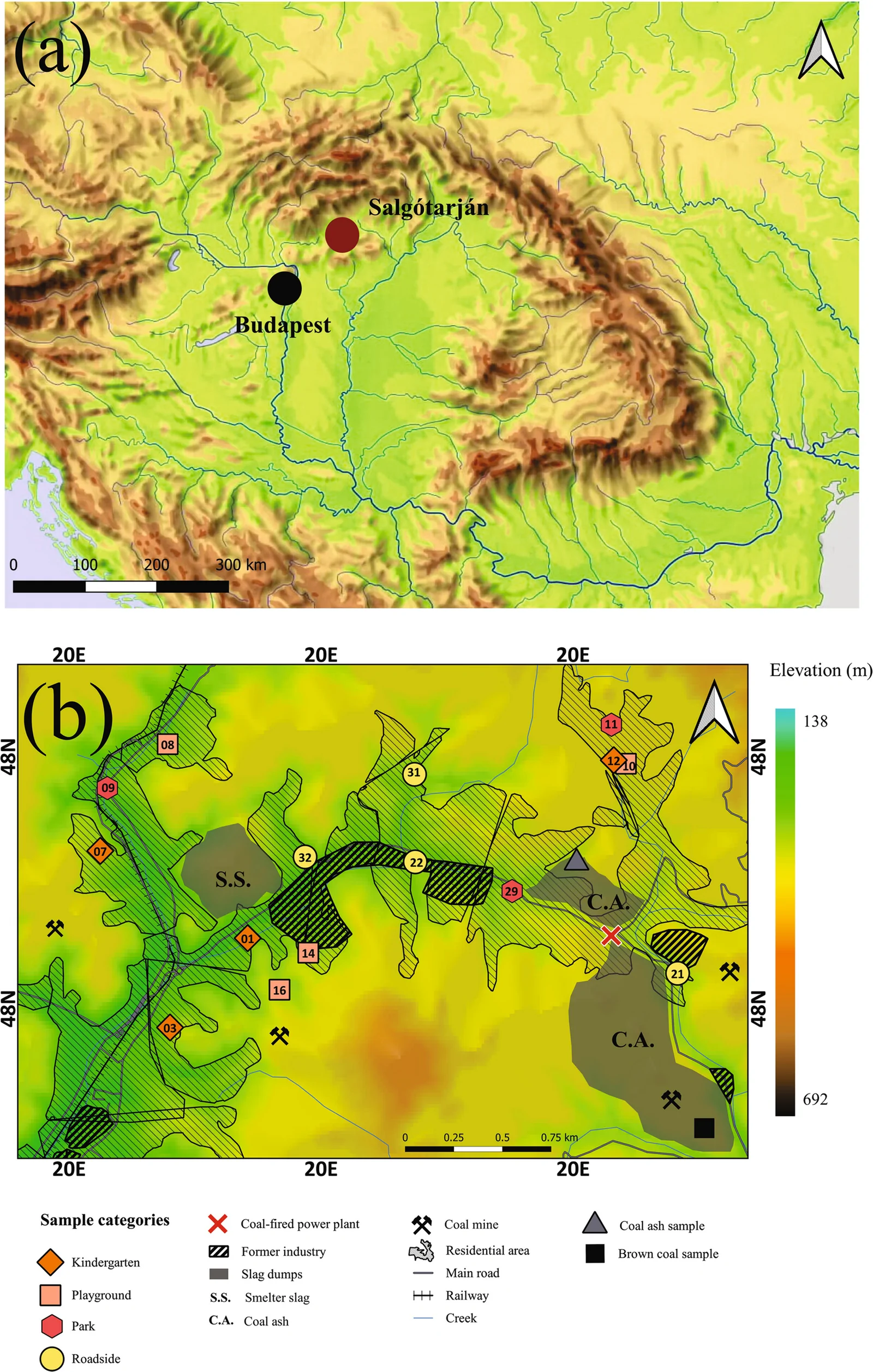
**a** Carpathian-Pannonian region showing the geographic environment of the Salgótarján city. **b** Map of the northeastern part of Salgótarján showing the studied urban soil sampling sites (*n* = 15) with categories, location of former industries, slag dumps, including smelter slag hill (S.S.), coal ash heaps (C.A.), as well as coal ash sample, brown coal sample, and coal-fired power plant are shown

Salgótarján city (48.08531˚N, 19.78689˚E) is located at an elevation of ~ 299 m above sea level in the northern part of Hungary, covering a total area of 102.96 km^2^ (Fig. 1a), with a recent population of ~ 35,000. The major geological formations of the studied area are covered by Neogene andesite, basalt, and different sandstones, siltstones, rarely rhyolite tuff, and extended brown coal deposit (Budai et al. 2015). The region features a hilly landscape with an annual average temperature of 8–9 °C, 500–550 mm of average annual precipitation, and an average annual sunshine duration of 2000 h (Gercsák et al. 2021). The dominant soil type in the region is brown forest soil (Pásztor et al. 2018).

### Sampling strategy

Fifteen urban soil samples were selected from a larger urban soil sample collection previously studied by Abbaszade et al. (2023), across the northeastern part of Salgótarján, based on their proximity to former industrial sites (CFPP, iron and steel works) (Fig. 1b). The samples were collected at a depth of 5 to 15 cm (the upper 5 cm soil was removed due to vegetation residues), following the Euro-Geo-Surveys sampling protocol (Demetriades et al. 2015). All collected samples represent different land-use types across Salgótarján, including kindergartens, playgrounds, parks, and roadsides. Additionally, a local brown coal sample representative of the type of coal used in the coal-fired power plant and a local coal ash sample representative of its combustion by-product, as studied by Angyal et al. (2004), were collected to support the identification of potential contamination sources. Details of the sampling strategy can be found in Abbaszade et al. 2023 and references therein.

### Methods

#### Sample preparation

All collected urban soil samples were air-dried at 20–22 °C for 14 days, following standard soil pretreatment protocols (ISO 11464, 2006). This step stabilizes the samples for subsequent analyses by minimizing the microbial and chemical reactions that typically occur in field-moist soils (e.g., Erich and Hoskins 2011). Homogenized samples passed through steel sieve system (Retsch AS 200, Germany) with mesh sizes of 2 mm. Large plant parts and gravel-sized materials, bricks, earthworms, and roots were eliminated at the Lithosphere Fluid Research Laboratory, Eötvös Loránd University.

A microwave-assisted digestion method was employed to prepare samples for Sr isotopic analysis at the Institute of Radiochemistry and Radioecology at Pannonian University in Veszprém (Hungary). Initially, 0.5 g of the sample was treated with 6 ml of concentrated HNO_3_, 0.5 ml of concentrated HCl, and 3 ml of 46% HF. The mixture gradually heated up to 200 °C for 15 min. The resulting solution was then treated with 1 ml of HNO_3_ and 5 ml of distilled water in a Teflon dish. To this solution, 3 ml of HNO_3_ and 1 ml of HF (46%) were added, followed by heating and evaporation to dryness. This step was repeated to ensure the accuracy of the results. Subsequently, 5 ml of HNO_3_ was added to the solution, which was then heated for an hour at 80 °C, and then diluted with 10 ml of distilled water. The solution was filtered after further heating for 30 min at 80 °C to complete the digestion process followed by Hegedűs et al. (2016).

#### Sr separation

After the complete digestion of samples, Sr was separated prior to its isotopic analysis at the Environmental Radionuclide Research Group in Fukushima, Japan. For separation of Sr from the solution, 0.5 mL of 100–150 μm particle size Sr extraction chromatography resin (Eichrom Technologies Inc, USA) was packed into polypropylene gravity columns (Muromac, Japan; size, 42 mm in length and 5 mm in diameter). The columns were rinsed with 10 mL H_2_O and preconditioned with 5 mL of 8 M HNO_3_ at a flow rate of 0.2 mL min^−1^. Each sample was dissolved in 5 mL of 8 M HNO_3_, and loaded onto the columns, followed by rinsing with 3 mL of 8 M HNO_3_ and 3 mL of 3 M HNO_3_. Finally, Sr was removed from the Sr resin using 3 mL of 0.05 M HNO_3_. This solution was evaporated to 0.1 mL and treated with 0.5 mL HNO_3_ and 0.5 mL H_2_O_2_. This mixture was boiled for 10 min and subsequently evaporated to dryness. The Sr-containing residue was then dissolved in 0.25 mL of 1 M HNO_3_ and evaporated to a tiny drop. This tiny drop of Sr was loaded onto a single degassed rhenium filament, along with one microliter TaF_5_ activator.

#### Analysis of ^87^Sr/^86^Sr isotope ratio

In this work, Phoenix X62 (Isotopx, UK) TIMS was used at the Environmental Radionuclide Research Group in Fukushima (Japan). For ^87^Sr/^86^Sr isotope ratio analysis, the multi-dynamic measurement procedure was applied, wherein the magnetic field settings dynamically altered during a measurement cycle, whereas the Faraday cup positions remained fixed (Di et al. 2021). Rubidium-85 was also monitored for isobaric interference correction caused on ^87^Sr. Considering the mass-dependent isotope fractionation of Sr during the thermal ionization process, the measured isotope ratios were normalized by 0.1194 (^86^Sr/^88^Sr). For quality control, the NIST‑SRM‑987 strontium carbonate isotopic standard (certified ^87^Sr/^86^Sr = 0.71034 ± 0.00026; NIST, 2007) was analyzed alongside the samples following the method described by Kavasi and Sahoo (2024). Analytical performance, including internal precision (2SE) and external precision (2SD), is reported in Table S1.

#### Analyses of bulk urban soil chemistry

Chemical analysis of bulk samples, including (Sr, P, and S), was performed using quadrupole-based inductively coupled plasma mass spectrometry (ICP-MS) with a detection limit of 0.01 mg kg^−1^, after modified aqua regia (1:1:1 HNO_3_: HCl: H_2_O) digestion in all urban soil samples performed at Bureau Veritas Global Company in Canada. To assess analytical performance, the STD DS11 certified reference material (CRM) was analyzed. The analytical accuracy, expressed as relative bias (RB %), was 1.34%, 2.71%, and 4.76% for Sr, P, and S, respectively, whereas the analytical precision, expressed as relative standard deviation (RSD %), was 5.72%, 4.17%, and 3.70%, indicating a high level of measurement quality (Table S2).

#### Grain size analysis

To determine the grain size fraction of the urban soil samples, 0.5 g of soil mixed with Na-pyrophosphate (Na_4_P_2_O_7_) and subjected to an ultrasonic cleaner. After standing overnight, the mixture was analyzed with a HORIBA PARTICA 950-V2 LA laser scattering particle size distribution analyzer at the Research and Industrial Relations Center (ELTE RIRC), Eötvös Loránd University. The particles were classified into three size fractions: < 8 μm (clay), 8–63 μm (silt) and > 63 μm (sand) (e.g., Beuselinck et al. 1998; Konert and Vandenberghe 1997; Thomas et al. 2021).

#### Soil properties

The determination of the organic matter content of the urban soil samples, including total organic carbon, and total nitrogen, was conducted at the Institute for Soil Sciences and Agricultural Chemistry in Hungary. Total organic carbon content was analyzed using the Hargitai/Tyurin method (Molnár et al. 2019), and total nitrogen was determined using the Kjeldahl method (Jr Conklin 2014). For pH measurement, soil samples were mixed with distilled water at a 1:10 soil-to-water ratio and shaken for 30 min. The pH was then measured in a 1:10 soil-water ratio using a digital Eijkelkamp 18.52.01 multimeter device at the Lithosphere Fluid Research Laboratory, Eötvös Loránd University.

#### Data analysis and mapping

Statistical analyses were conducted using OriginPro 2024 (v10.1.0.170, OriginLab Corporation, Northampton, MA, USA). The Shapiro-Wilk test, which is the appropriate statistical test for small sample sizes (*n* < 50) (Shapiro and Wilk 1965), confirmed normality (*p* > 0.05) for Sr concentrations and isotopic compositions across 15 urban soil samples. Linear regression analysis was applied to examine potential relationships between Sr concentrations and isotopic ratios. Sampling locations (Fig. 1b) were recorded by GPS and geospatial processing and mapping were performed using QGIS (v3.26.3, Open-Source Geospatial Foundation). The spatial distribution of Sr concentration in urban soils was prepared using graduated map based on manual classification.

A binary mixing (two-end-member) model (Deng et al. 2021; Lin et al. 2016; Zhao et al. 2019) was used to semi-quantitatively estimate relative contributions of coal ash vs. local soil (Eqs. 1 and 2). This estimation is defined as semi-quantitative because it simplifies a complex urban environment, which likely contains multiple additional anthropogenic sources (such as historical smelter slag), into a two-component system. Consequently, the calculated contribution percentages are not intended to represent absolute, highly precise source apportionment results, but rather serve as trend-based, indicative findings to effectively illustrate the relative spatial influence of coal ash across the sampling sites.

1 $$Sr_{coal\;ash}\left(\%\right)=\frac{\left({}^{87}Sr/{}^{86}Sr\right)sample-\left({}^{87}Sr/{}^{86}Sr\right)local\;soil}{\left({}^{87}Sr/{}^{86}Sr\right)coal\;ash-\left({}^{87}Sr/{}^{86}Sr\right)local\;soil}$$

2 $$Sr_{local\;soil}\left(\%\right)=100\%-Sr_{coal\;ash}\left(\%\right)$$

where the two end-members represent the ^87^Sr/^86^Sr of coal ash derived from coal-fired power plant (the primary contamination source in the study area), and the ^87^Sr/^86^Sr of the local soil which is not contaminated by coal ash, representing the urban soil matrix (Fig. 1b).

## Results and discussion

### Strontium distribution in urban soils

The concentration of Sr in urban soil samples from Salgótarján ranges from 17.5 to 51.3 mg kg^−1^, with a mean value of (30.6 ± 10.3 mg kg^−1^, *n* = 15) (Table 1). The spatial distribution of Sr (Fig. S1) shows elevated concentration in the urban soil samples collected adjacent to coal-fired power plant (CFPP), slag dumps and former industrial sites (i.e., iron and steel works). Among the five urban soils with the highest Sr concentrations, four sampling sites, STN29 (park), STN21 (roadside), STN14 (playground), and STN16 (playground), have Sr concentrations of 51.3 mg kg^−1^, 48.6 mg kg^−1^, 37.5 mg kg^−1^, and 35.8 mg kg^−1^, respectively (Table 1, Fig. 1b). These sites are located at distances less than 3 km to the CFPP (Table 1, Fig. 1b). Nevertheless, the sample site STN09 (park), located at distance of 4.4 km from CFPP has even the third highest Sr concentration (43.1 mg kg^−1^) (Table 1, Fig. 1b).

**Table 1:** The concentration of strontium and ^87^Sr/^86^Sr isotopic ratios in collected urban soil samples from Salgótarján: categories and locations. Physicochemical properties: grain size (clay, silt, sand), total nitrogen (TN), total organic carbon (TOC), phosphorus (P), and sulfur (S) values are presented in (m/m) %. (Data for grain size, TN, TOC, and pH adapted from Abbaszade et al. 2023)

Sample ID	category	Sr (mg kg^−1^)	^87^Sr/^86^Sr	P (m/m%)	S (m/m%)	TN (m/m%)	TOC (m/m%)	pH	Clay (m/m%)	Silt (m/m%)	Sand (m/m%)	Distance from the CFPP (km)	Long (E)	Lat (N)
STN 01US	Kindergarten	30.7	0.716405	0.063	0.04	0.13	1.39	7.54	20.2	58.6	21.3	3.0	19.819486	48.111610
STN 03US	Kindergarten	23.4	0.716890	0.039	0.05	0.19	1.76	7.62	19.2	57.1	23.7	3.7	19.810965	48.105041
STN 07US	Kindergarten	18.7	0.717330	0.033	0.02	0.10	0.87	7.40	32.4	50.6	17.0	4.3	19.803305	48.118028
STN 12US	Kindergarten	25.3	0.711521	0.065	0.03	0.21	1.64	7.25	14.1	58.8	27.1	1.4	19.859678	48.124699
STN 08US	Playground	24.9	0.715630	0.054	0.03	0.19	1.61	7.31	28.7	53.3	18.0	4.0	19.810702	48.125876
STN 10US	Playground	29.2	0.714581	0.068	0.03	0.14	1.24	7.62	10.1	40.0	49.9	1.4	19.861010	48.124438
STN 14US	Playground	37.5	0.715711	0.095	0.10	0.21	1.69	6.61	13.4	45.9	40.8	2.5	19.826140	48.110543
STN 16US	Playground	35.8	0.710303	0.043	0.05	0.09	0.78	7.55	19.6	57.8	22.6	2.8	19.822983	48.107819
STN 09US	Park	43.1	0.713844	0.063	0.26	0.21	1.66	5.86	16.5	52.9	30.7	4.3	19.804080	48.122652
STN 11US	Park	17.5	0.718300	0.072	0.04	0.25	1.82	7.46	15.2	52.5	32.4	1.7	19.859420	48.127304
STN 29US	Park	51.3	0.713573	0.060	0.09	0.21	2.55	7.30	20.7	50.4	28.9	0.9	19.848522	48.115076
STN 21US	Roadside	48.6	0.713175	0.062	0.06	0.23	2.28	7.52	4.0	38.6	57.3	0.6	19.866687	48.109024
STN 22US	Roadside	18.7	0.718107	0.052	0.03	0.20	1.58	7.34	10.7	50.9	38.4	1.8	19.837885	48.117223
STN 31US	Roadside	25.7	0.719559	0.091	0.03	0.13	0.96	7.45	12.7	37.0	50.3	2.1	19.837806	48.123656
STN 32US	Roadside	27.9	0.711573	0.068	0.04	0.14	1.47	7.43	14.6	49.4	36.0	2.6	19.825789	48.117586
Mean ± SD		30.6 ± 10.3	0.715100 ± 0.002750	0.062 ± 0.016	0.06 ± 0.06	0.18 ± 0.05	1.55 ± 0.46	7.28 ± 0.45	16.81 ± 6.90	50.25 ± 6.85	32.96 ± 11.96			
STN CA	Coal ash	113.6	0.711652	0.021	0.57	-	-	-	10.8	26.8	62.3	0.5	19.852833	48.120688
STN BC	Brown coal	52.8	0.712830	0.003	0.43	-	-	-	-	-	-	0.4	19.873072	48.097101

The brown coal (BC) sample from Salgótarján has Sr concentration of 52.8 mg kg^−1^ (Table 1), which is derived from the Neogene brown coal^1^ formations characteristic of Hungary (Landis et al. 2004). Similarly, the Sr content in brown coal from Ózd^2^ (a former industrial city in Hungary, located in close distance northeast of Salgótarján), supplied from the same local mining area, is 66.1 mg kg^−1^. These values align with several studies, across different Central Eastern, and South European countries, reported the petrology and geochemistry of brown coal deposits (Table S3), containing trace elements, such as Sr, to better understand their behavior during coal combustion and subsequent environmental impact. For instance, Neogene brown coal deposits from central Slovakia show Sr concentrations ranging from 55.1 to 71.4 mg kg⁻^1^ (mean, 62.6 mg kg⁻^1^) (O’Keefe et al. 2011), falling Salgótarján Sr concentration within the range. The brown coal deposits (Upper Miocene age) from northeastern Serbia show Sr concentration (mean, 125 mg kg⁻^1^) (Životić et al. 2019) significantly higher than that of Salgótarján. The Sr concentration (mean, 95 mg kg⁻^1^) in southern Bulgarian Neogene brown coal (Late Miocene to Eocene age) (Eskenazy and Minčeva 1989) also exceeds the Sr concentration observed in Salgótarján (Table S3). Beyond these Neogene Central Eastern European brown coals, the brown coal deposits in central western Portugal (Pliocene age) show mean Sr concentration of 47 mg kg⁻^1^, slightly lower than that of Salgótarján (Suárez-Ruiz et al. 2006). Although all these samples have the same age (Neogene) and coal type (brown coal), their regional variations in Sr concentrations arise from different geological and environmental factors. According to aforementioned studies, the similarity in Sr concentrations of brown coal between Salgótarján and central Slovakia can be explained by their proximity within the Carpathian-Pannonian region and probably their similar carbonate (particularly calcite, CaCO_3_) content. Higher Sr levels in northeastern Serbia and southern Bulgaria can be attributed to increased carbonate, thus, elevated Sr concentration, whereas in the Portugal Neogene brown coal (Suárez-Ruiz et al. 2006) the opposite Sr behavior can be observed.

However, it is also notable that Sr concentrations of the burning coal increase significantly during the combustion in coal-fired power plants. During this high temperature process, due to the geochemical nature of Sr, it is participating and accumulating in by-products like slag and coal ash (e.g., Hower et al. 1998; Ruhl et al. 2014). As recently reported by Dai et al. (2025), the global mean Sr concentration in Neogene brown coal is 245 mg kg⁻^1^, whereas coal ash shows significantly higher mean value (740 ± 70 mg kg⁻^1^; Ketris and Yudovich, 2009 and references therein) than unburnt brown coal, highlighting the Sr enrichment during combustion process. A similar pattern is observed in Salgótarján, where the Sr concentration in brown coal is significantly lower than that in the coal ash sample collected from a coal ash cone (113.6 mg kg⁻^1^, located 0.5 km from CFPP; Fig. 1b, Table 1). It is also consistent with Sr concentration in coal ash from combustion of the Neogene coal in southern Greece, ranges from 375 to 1345.3 mg kg⁻^1^, with some values exceeding 900 mg kg⁻^1^ (Gentzis et al. 1996).

The accumulation of Sr in coal ash during combustion is primarily influenced by addition of limestone (CaCO_3_) and other Ca-rich sorbents during coal burning process (see details in the “Variations of phosphorus and sulfur content” section). During this process, Sr easily substitutes Ca in Ca-bearing minerals due to these lithophile elements similar geochemical properties, facilitating its incorporation into the coal ash (Bragança and Castellan 2009; Turekian and Kulp 1956). The presence of coal ash in the urban environments, particularly around coal-fired power plants, can significantly influence the Sr concentration in urban soils with local variations depending on factors such as distance from the emission source, and human disturbance. The studied Salgótarján urban soil samples with the highest Sr concentrations, such as STN29 (park), and STN21 (roadside) are located near the CFPP (Table 1, Fig. 1b). However, variations in Sr distribution across the studied urban soil samples, for example the elevated Sr concentration observed in STN09 (park) (Table 1, Fig. S1), despite its greater distance from CFPP, suggesting additional factors beyond proximity to CFPP influence Sr levels in certain sample sites. Comparative studies around the world highlighted the impact of brown coal combustion by-products (i.e., coal ash) on Sr concentrations in urban soils (Table S4). For instance, the surface soil samples of Bitola region (Macedonia), show Sr concentrations ranging from 3.6 to 420 mg kg⁻^1^ (mean: 89 mg kg⁻^1^), correlated with emission from coal thermoelectric plant, burning brown coal similar to Central Eastern European Neogene formations (Stafilov et al. 2018). In West Bengal (India), Sr concentrations in surface soils (mean, 56.8 ± 4.3 mg kg⁻^1^) near coal-fired stations, reflecting fly ash deposition from CFPP emissions (George et al. 2015). The Sr concentration in surface soil samples collected from Inner Mongolia (China), ranging from 148 to 201 mg kg⁻^1^ (mean: 172 ± 17 mg kg⁻^1^), reflecting significant contribution of coal combustion residues to the local environment (Gong et al. 2018). In the United States, soils collected near Savannah river (Georgia) coal-fired power plants show Sr concentrations up to 60 mg kg⁻^1^, in the soil samples collected rather closer to the emission source (Evans et al. 1980). However, an exception is observed in Setúbal city (Portugal) coal type, where bituminous coal was used for combustion, with similar combustion technique, resulted in comparable dataset. In the mentioned study, surface soils exhibit Sr concentrations ranging from 20 to 299 mg kg⁻^1^ (mean, 95 ± 91 mg kg⁻^1^), attributed to decades of coal ash deposition from a nearby coal-fired plant (Dinis et al. 2021). These findings align with observed impact of coal ash deposition on Sr distribution across studied Salgótarján urban soils (Fig. S1). This highlights the significant role of proximity to emission sources, such as coal-fired power plants, in determining Sr concentrations in urban soils of the surrounding environment, as well as emphasizing the need to consider local environmental factors to better understand Sr variability.

### Physicochemical properties, P, and S content of soil and their connection with Sr concentration

#### Variation of pH in urban soils

The Salgótarján urban soil samples are predominantly weakly alkaline (mean pH, 7.28 ± 0.45), reaching a maximum of 7.62 at STN03 (kindergarten), and STN10 (playground) (Table 1, Fig. 1b). However, only two samples, STN09 (park) and STN14 (playground), fall below pH = 7, with lower pH values of 5.86 and 6.61, respectively (Table 1, Fig. 1b). Elevated pH observed in samples STN03 (kindergarten, pH 7.62), STN07 (kindergarten, pH 7.40), and STN11 (park, pH 7.46), with particularly low Sr concentrations (Table 1, Fig. S1), can be due to the great contribution of artificial infills into the surface soil during urbanization and recent landscape changes. Such infills are predominantly made from common silicate minerals (like quartz, feldspar, mica, etc.) (Fig. S2), introduced for site leveling and foundation stabilization during urban development, which has been reported as a common practice in recent studies on urban soils in Hungary by Puskás et al. (2008), and Horváth et al. (2014). In contrast, samples with elevated pH, such as STN29 (park, pH: 7.30), STN21 (roadside, pH: 7.52), and STN16 (playground, pH: 7.55), which show distinctly high Sr concentrations (Table 1, Fig. 1b), correspond to significant coal ash content (Fig. S3), as coal ash of the brown coal combustion is highly alkaline due to its high Ca (and Mg) content (e.g., Gaffney and Marley 2009; Kolbe et al. 2011). However, the reduced pH values in samples STN09 (park): 5.86, and STN14 (playground): 6.61, with high Sr concentrations (Table 1, Fig. 1b), reflect the influence of their proximity to the smelter slag hill and the former iron and steel factory, respectively (Fig. 1b). This is explained by the formation of secondary minerals like gypsum and iron (oxy-)hydroxides in smelter slags through chemical weathering over decades, which reduces the pH level (Riley et al. 2024; Sueoka and Sakakibara 2013).

#### Variations of phosphorus and sulfur content

In the Salgótarján urban soils, P content varies from 0.033 m/m % (STN07: kindergarten) to 0.095 m/m % (STN14: playground), with a mean of 0.062 ± 0.016 m/m %, whereas S content ranges from 0.02 m/m % (STN07: kindergarten) to 0.26 m/m % (STN09: park), with a mean of 0.06 ± 0.06 m/m % (Table 1). Phosphorus in coal ash primarily originating from phosphate minerals (mostly apatite: Ca_5_(PO_4_)_3_(OH,F,Cl)), which can enrich the P level to 0.1–0.5 m/m %, significantly higher than its concentrations in raw coal (e.g., Vamvuka et al. 2005; Qafleshi et al. 2013; Bielowicz 2020). During combustion, apatite is produced as a secondary mineral in coal ash due to reactions between volatile P compounds, such as P_2_O_5_ (showing highly hygroscopic and microcrystalline character), and available Ca, which condense onto fly ash particles. In addition, during this process, the relict of stable phosphate compounds (like calcium orthophosphates (Ca_3_(PO_4_)_2_), elevates the P concentration in the coal ash, typically reaching ranges between 0.5 m/m % and 1.5 m/m % (Mishra et al. 2016; Zhang and Ninomiya 2007). Similarly, S in coal ash predominantly occurs as sulfate minerals, like gypsum (CaSO_4_). Gypsum forms through the reaction of Ca from additive Ca-rich sorbents (as discussed earlier) with sulfur gases (SO_2_) released during coal combustion (known as desulfurization process) (e.g., Spivak-Birndorf et al. 2012). In agreement with these findings, the Salgótarján coal ash sample shows elevated concentrations of both P (0.021 m/m %), and S (0.57 m/m %) compared to STN brown coal containing (0.003 m/m % P and 0.43 m/m % S; Table 1). These differences highlight the enrichment of P and S during the combustion process, as coal ash contains P in phosphate and S in sulfate minerals. However, in urban environments additional sources may also contribute to elevated P and S contents. The highest P-content, 0.095 m/m % in (STN14: playground) (Table 1, Fig. 1b) even exceeds the P content of the coal ash by more than four times. Although this enrichment is likely driven by local phosphate enrichment from coal ash deposits, which can contain high P (0–5 m/m %) reported, for instance in Dawson and Siegwolf. (2007), additional urban inputs cannot be ruled out. For instance, during steelmaking process, P was added in the form of apatite or phosphorite to the molten Fe to increase the fluidity of the melt for better castability (Turner and Roberts-Austen 1908). Therefore, the resulting by-product (i.e., smelter slag) have significantly elevated concentrations of P, with reported ranges typically ranging ~ 3–6 m/m % (Yu et al. 2023). Due to its high phosphate content, this slag was a major source of P for agricultural purposes, which could be utilized as a valuable fertilizer for local farmers, a common practice for agricultural purposes reported in several studies (e.g., ITO, 2015; Deus et al. 2018; Tozsin and Öztaş, 2023). Furthermore, in recreational areas like playgrounds, phosphorus levels may be further augmented by anthropogenic activities, such as contemporary application of NPK fertilizers or the accumulation of urban organic waste (e.g., Lorenz and Lal 2009). Similarly, the highest S-content, 0.26 m/m % in STN09 (park) (Table 1, Fig. 1b), suggesting significant sulfate input not only from coal ash but also from smelter slag deposition. Historically, the local brown coal was used as the primary energy source for the blast furnace, along with the Fe ore. Therefore, this peak may also reflect the combined effects of historical atmospheric sulfate accumulation and the legacy of SO_2_ emissions from local coal combustion, as well as iron and steel industrial processes in Salgótarján, which likely contributed to long-term sulfur sequestration in the soil matrix via dry and wet deposition (e.g., Fowler et al. 2007). In contrast, STN07 (kindergarten) has the lowest P (0.033 m/m %) and S (0.02 m/m %) values, (Table 1, Fig. 1b), consistent with its significantly low Sr concentration (18.7 mg kg^−1^) (Table 1, Fig. 1b), indicating minimal influence from coal ash and/or smelter slag deposits, as well as additional anthropogenic inputs during landscape and land use changes.

#### Variation of TOC and TN content

The total nitrogen (TN) and total organic carbon (TOC) contents vary significantly among the studied Salgótarján urban soil samples: TN ranges from 0.09 m/m % (STN16: playground) to 0.25 m/m % (STN11: park) with mean value of 0.18 ± 0.05 m/m %, whereas TOC ranges from 0.78 m/m % (STN16: playground) to 2.55 m/m % (STN29: park) with mean value of 1.55 ± 0.46 m/m % (Table 1, Figure 1b). These values reflect the significant variation in organic matter content based on different land-use types and their proximity to pollution sources (e.g., Abbaszade et al. 2023; Hiller et al. 2015). Furthermore, climate and microbial activity influence organic matter cycling and stabilization, making high variation in TOC, and TN content of soil across different environments, recently studied by Chatterjee et al. (2024), and Pereira et al. (2021). The TOC and TN mean values in the studied Salgótarján urban soils are lower than those reported for urban soils worldwide (mean values: TOC 6.54 m/m %, TN 0.41 m/m %) (Pouyat et al. 2015), highlighting the role of urban management, landscape changes, vegetation cover and anthropogenic inputs on the organic matter content of the urban soil.

**Fig. 2:**
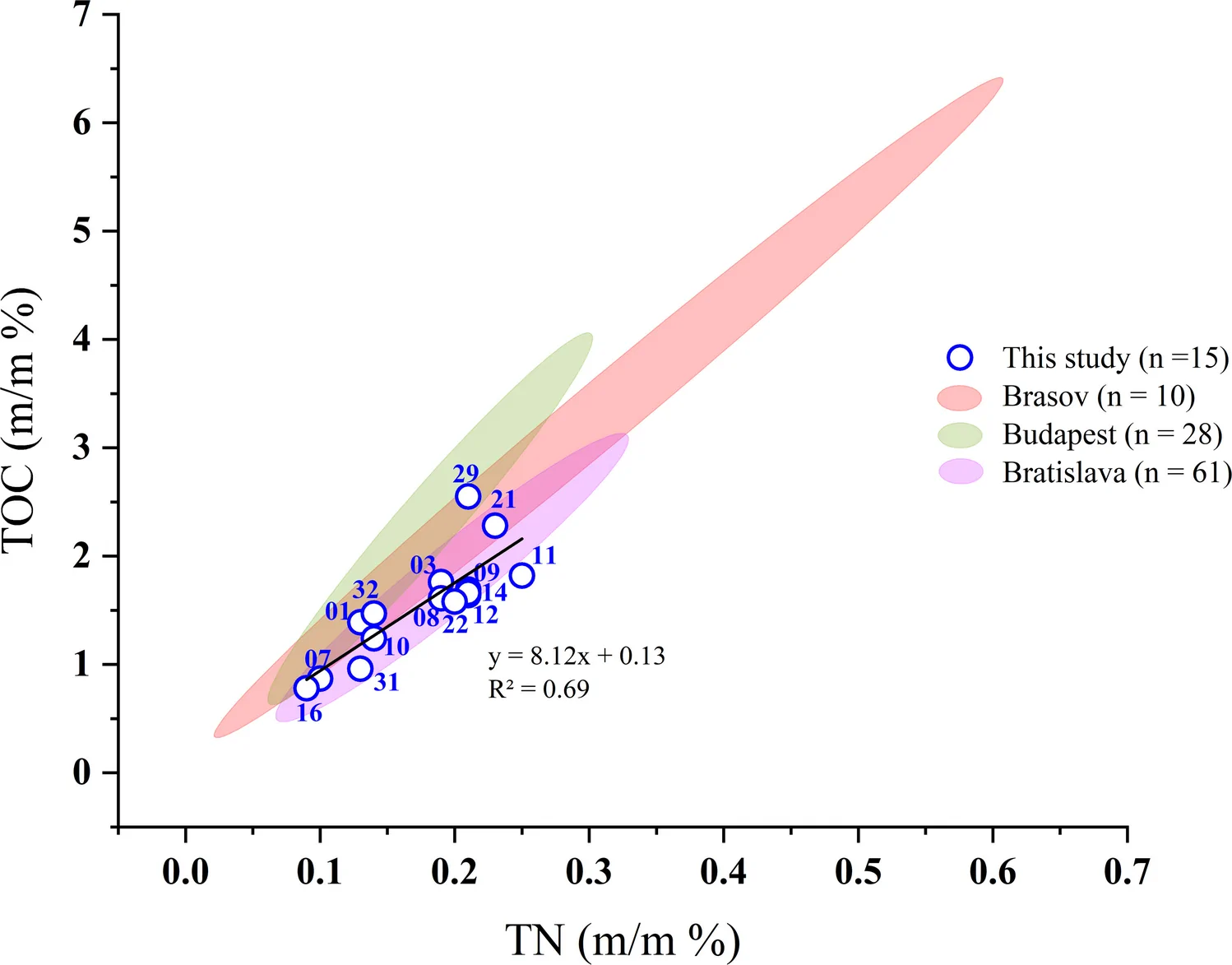
Variations of TOC vs. TN in Salgótarján urban soils in comparison with those from Central-Eastern Europe. Blue open circles represent data points from Salgótarján urban soils. The pale red area indicates Brasov urban soils (Enescu et al. 2022), green area indicates Budapest urban soils (Mónok et al. 2021), and purple area shows Bratislava urban soils (Hiller et al. 2015). Values of the comparative data are given as maximum, minimum, and mean (if any)

Interestingly, samples with elevated TOC and TN, such as STN29 (park, TOC 2.55 m/m %, TN 0.21 m/m %), and STN21 (roadside, TOC: 2.28 m/m %, TN 0.23 m/m %), also exhibit high Sr concentrations of 51.3 mg kg⁻^1^ and 48.6 mg kg⁻^1^, respectively (Table 1, Fig. 1b), suggesting a potential association between organic matter and Sr content, which results in its stabilization in the soil matrix (e.g., Gupta & Walther 2017; Sufian 2021). In contrast, STN16 (playground), with the lowest TOC (0.78 m/m %) and TN (0.09 m/m %), shows relatively high Sr content (35.8 mg kg⁻^1^, even higher than Salgótarján Sr mean: 30.6 ± 10.3 mg kg⁻^1^) (Table 1, Fig. 1b), proving that Sr accumulation in this sample is not solely governed by soil organic matter but also by anthropogenic inputs like coal ash and/or smelter slag as was described above in the previous chapter. In comparison of TN and TOC contents in Salgótarján urban soils (Table 1) with other Central-Eastern European studies (Fig. 2), regional variability is evident. To ensure consistency in comparison data, missing TOC or TN values in urban soils of Brasov (Romania) (Enescu et al. 2022), and Bratislava (Slovakia) urban soils (Hiller et al. 2015), were calculated using a C/N ratio of 10 g C g⁻^1^N, which represents the lower range typical for temperate soils in Central-Eastern Europe (10–30 g C g⁻^1^N), including Salgótarján (Cools et al. 2014; Nadelhoffer et al. 1999). Urban soils in Brasov exhibit broader TN (0.04–0.66 m/m %, mean 0.28 m/m %) and TOC (0.37–6.6 m/m %, mean 3.49 m/m %) ranges (Fig. 2) than those of Central Eastern European regions including Salgótarján, attributed to natural humiferous soil. In contrast, narrower ranges observed in Bratislava (Slovakia) kindergartens and playgrounds (TN 0.08–0.32 m/m %, mean 0.20; TOC 0.79–3.23 m/m %, mean 1.79) and Budapest urban soils (TN 0.07–0.31 m/m %, mean 0.17; TOC 0.85–4.34 m/m %, mean 2.21) (Fig. 2), reflect urbanization impacts, such as vegetation removal, soil compaction, by using artificial infills. These patterns align with Salgótarján urban soils, particularly playgrounds and kindergartens, where soil management practices and limited organic inputs significantly influence TN and TOC content. However, elevated TOC and TN in Salgótarján parks (e.g., STN29, and STN09) are linked to high vegetation cover and organic inputs (Fig. S4). According to recent studies (e.g., Stephanou et al. 2021; Wang et al. 2017), the low TN content in the urban soils corresponds to reduced nitrogen cycling due to land-use changes and diminished microbial diversity. Additionally, microbial activity studies in Salgótarján urban soils reported by Abbaszade et al. (2023) found reduced microbial activity, particularly in STN09 (park) and STN14 (playground), linked to anthropogenic inputs, such as As, Pb, Hg, and Cd from coal ash and smelter slag, as further observed in our study by the elevated Sr concentrations at these sites (Table 1, Fig. 1b), characteristic of high inputs of industrial by-products.

#### Grainsize distribution

**Fig. 3:**
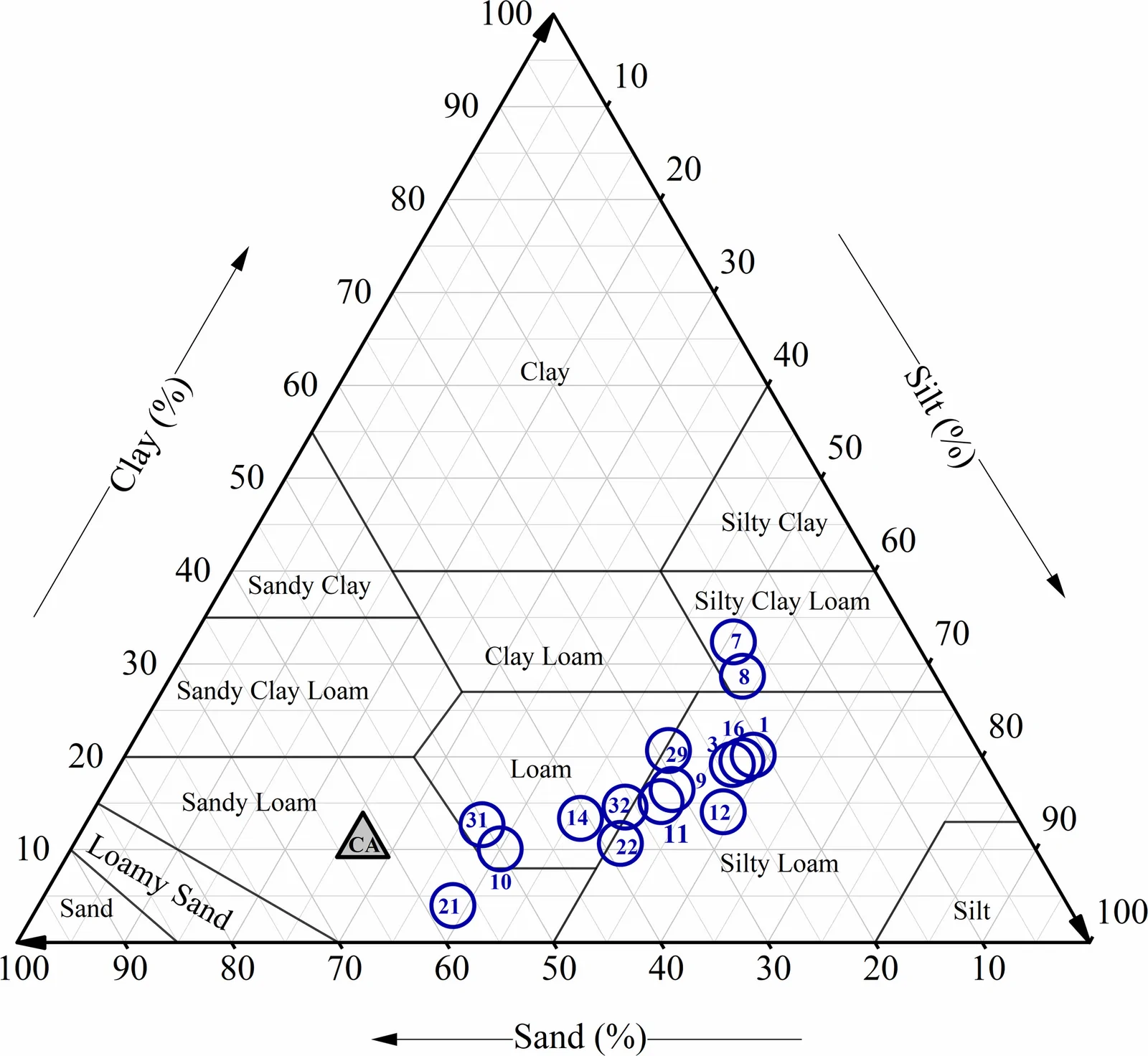
Soil textural triangle of the studied Salgótarján urban soils using the USDA-NRCS (1993) soil texture classes. Blue open circles with numbers mark the studied urban soil samples, and gray triangle marks coal ash sample from the coal ash cone (corresponding additional data in Table 1)

According to the United States Department of Agriculture - Natural Resources Conservation Service (USDA-NRCS) soil texture classification (Staff 1993), most of the studied STN urban soils exhibit a granulometric distribution characteristic of sandy loam to silty-clay-loam textures (Fig. 3, Table 1). Furthermore, the clay grain size fraction ranges from 4.0 m/m % (STN21: roadside) to 32.4 m/m % (STN07: kindergarten). The silt grain size fraction shows the highest percentage (58.8 m/m %), in STN12 urban soil (kindergarten), whereas sample STN31 (roadside) has the lowest value of 37.0 m/m %. The sand grain size fraction ranges from 17.0 m/m % (STN07: kindergarten) to 57.3 m/m % (STN21: roadside) (Fig. 3, Table 1). This variability across studied urban soils is likely attributed to the introduction of artificially made soils^3^ (mainly composed of quartz dominated materials) (Fig. S2) during land cover changes, as well as addition of organic materials (e.g., turfgrasses) for stabilizing the surface soil, a common practice during past urbanization and landscape changes (e.g., Dercon et al. 2005).

The coal ash sample shows the highest sand grainsize fraction (62.3 m/m%), exceeding the urban soil range, comparable to global findings on coal ash grain size distributions (e.g., Halânga, Romania: 65.7 m/m% (Ghermec et al. 2015); India: 72.5% (Kumar et al. 2014); Tanjung Bin, Malaysia: similar to sand grain size fraction (Ramzi et al. 2016)).

The grain size distribution in STN07 (kindergarten) and STN08 (playground) urban soils indicates silty-clay-loam texture (Fig. 3, Fig. S5). Despite their fine grain sizes, these soils exhibit relatively low Sr concentrations (18.7 mg kg⁻^1^ for STN07 and 24.9 mg kg⁻^1^ for STN08; Table 1, Fig. 1b), suggesting limited coal ash content and predominance of geogenic Sr (Querol et al. 1995). In contrast, STN31 (roadside), with a sandy texture and Sr concentration of 25.7 mg kg⁻^1^, likely reflects structural incorporation within carbonate phases present in additive materials of urban infrastructure (e.g., artificial infills and road-base aggregates). However, high Sr concentration (35.8 mg kg⁻^1^) in STN16 (playground; Table 1, Fig. 1b), with a silty texture, reflects improved adsorption capacity, facilitating greater contaminant accumulation (Davis et al. 2001). This underscores the role of finer grains in accumulating pollutant content due to their higher specific surface area and sorption capacity.

### ^87^Sr/^86^Sr isotopic ratios in urban soils

#### Spatial trends and variability in ^87^Sr/^86^Sr ratios

The ^87^Sr/^86^Sr ratios of the studied urban soil samples fall within the range of 0.710303–0.719559 with a mean value of 0.715100 ± 0.002750 (Table 1). The highest ^87^Sr/^86^Sr ratio is observed in sample STN31 (roadside), located on the northern side of Salgótarján at the edge of city, near a meadow area used for livestock grazing, whereas sample STN16 (playground) shows the lowest ^87^Sr/^86^Sr ratio, collected close to area of the former iron and steel work (Fig. 1b).

**Fig. 4:**
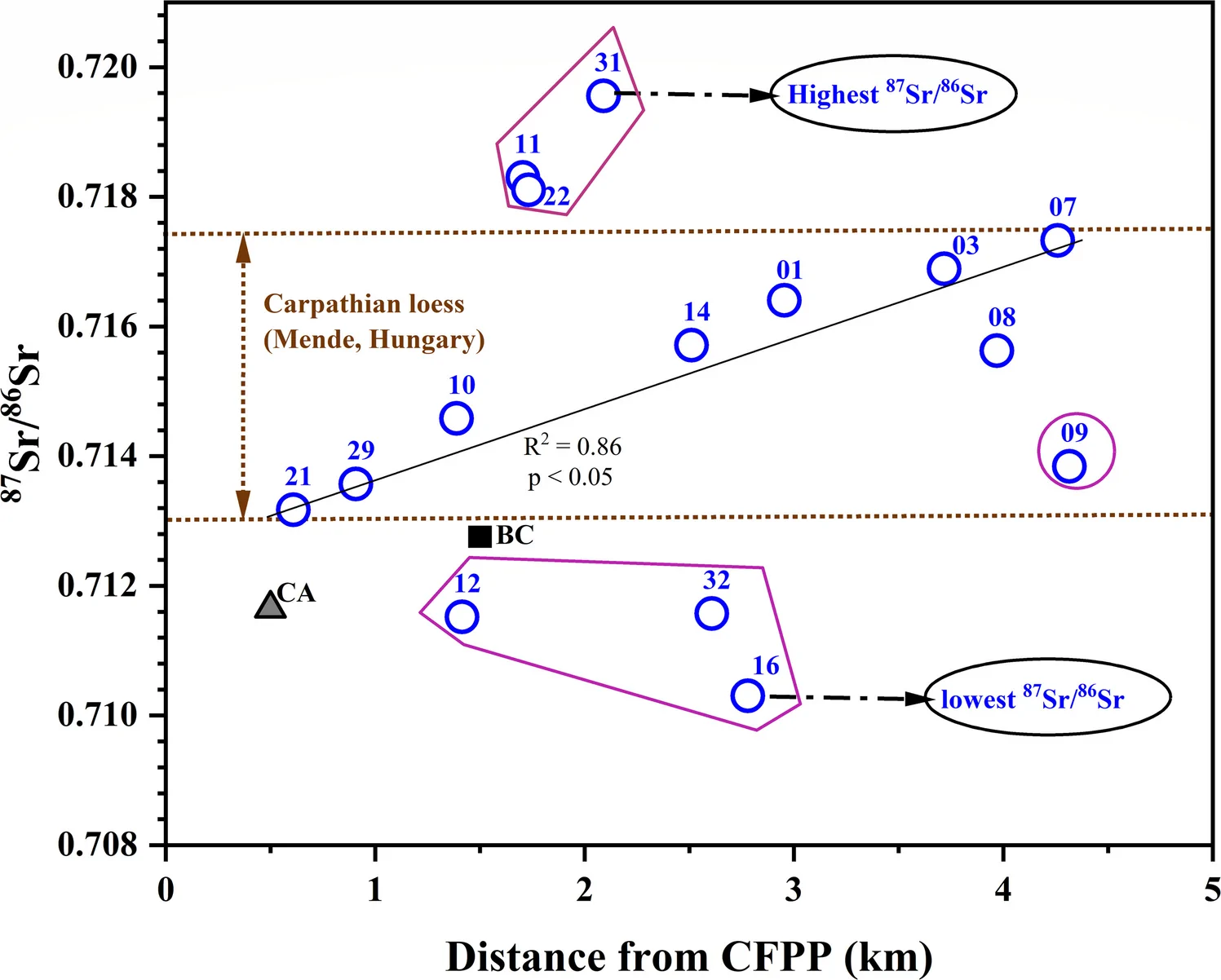
Variation of ^87^Sr/^86^Sr ratio in Salgótarján urban soil, coal ash, brown coal samples in function of sampling site distance from the Salgótarján coal-fired power plant (CFPP). Blue open circles with numbers indicate the studied urban soil samples, gray closed triangle and black closed square mark coal ash (CA) and brown coal (BC) samples, respectively. The regression line (*R*^2^ = 0.86, *p* < 0.05) shows the positive correlation observed for the subset of urban soil samples with ^87^Sr/^86^Sr values within the range reported for Carpathian loess (e.g., Mende, Hungary; 0.713162–0.717517; Ujvári et al. 2012), indicated by the brown dotted lines. Samples that deviate from this trend are highlighted in pink polygons (clusters 11‑22‑31 and 12‑16‑32, and sample 09)

A clear trend is observed in more than 50% of the studied urban soil samples, including STN21 (roadside), STN29 (park), STN10 (playground), STN14 (playground), STN01 (kindergarten), STN03 (kindergarten), STN08 (playground), and STN07 (kindergarten), which exhibit a positive correlation (*R*^2^ = 0.86, *p* < 0.05) between the increasing ^87^Sr/^86^Sr ratio as a function of increasing distance from the former CFPP (Fig. 4). These samples exhibit ^87^Sr/^86^Sr values consistent with variable contributions from local soil material and coal ash (Fig. 4). In contrast, the remaining samples (above the trend: STN11, STN22, STN31; below the trend: STN12, STN16, STN32, and STN09; Fig. 4) deviate from this trend, indicating that additional sources should be considered as contributors to their isotopic signatures.

#### Coal ash influence on low ^87^Sr/^86^Sr ratios in urban soils

The coal ash sample (CA), derived from the closest distance to the CFPP (0.5 km), is characterized by low ^87^Sr/^86^Sr ratio of 0.711652 (Table 1, Fig. 4). This low data for coal ash is consistent with those from USA coal ash (e.g., Mattigod et al. 1990; Petrović & Fiket 2022) showing low Sr isotopic ratios (range 0.70723–0.70837, mean 0.70767 ± 0.00057; Mattigod et al. 1990), (range 0.7097–0.7112, mean 0.7106; Petrović & Fiket 2022). This can be explained by two major factors. First, Sr in coal largely derives from carbonates within the coal seams, typically of marine origin, such as calcite-rich layers. These marine limestones exhibit globally similar isotopic ratios, with a range of 0.7080–0.7090 (Prokoph et al. 2008), due to the long residence time of Sr in seawater and low ^87^Rb content. Second, additive material (i.e., limestone), which are generally used in the combustion process (as explained earlier), can further shift isotopic ratios slightly above or below this range, as studied in details by Semikhatov et al. (2002). Consequently, the slightly higher ^87^Sr/^86^Sr ratio observed in the Salgótarján coal ash (0.711652) compared to aforementioned studies likely reflects the combined influence of the carbonates present in the local Neogene coal and the additives used during coal combustion.

Among the sample groups described in the previous section, the lowest ^87^Sr/^86^Sr ratios observed in the urban soil samples STN21 (roadside), STN29 (park), and STN10 (playground) with ^87^Sr/^86^Sr ratios of 0.713175, 0.713573, and 0.714581, respectively, which are closer to the CFPP at 0.6, 0.9, and 1.4 km (Table 1, Fig. 4), can be attributed to the significant influence of coal ash. At intermediate distances (~ < 3 km), STN14 (playground) and STN01 (kindergarten) show moderately higher ratios of 0.715711 and 0.716405, respectively, reflecting reduced influence of coal ash ^87^Sr/^86^Sr ratio, and moderate influence from local soil (e.g., natural soil/artificial infills) during land cover changes (Table 1, Fig. 4).

#### Local soil influence on high ^87^Sr/^86^Sr ratios

At distances beyond 3 km from CFPP, samples STN03 (kindergarten), STN08 (playground), and STN07 (kindergarten) show ratios of 0.716890, 0.715630, and 0.717330, respectively, consistent with minimal or negligible coal ash contribution, and dominant influence from local soil (natural soil/artificial infills, such as quartz-rich materials) (Table 1, Fig. 4). Notably, these urban soil samples, along with those discussed in the previous section, indicate ^87^Sr/^86^Sr ratios that fall within the range of 0.713162–0.717517, reported for the Mende loess (Hungary) (Fig. 4), which is relatively uniform across the Pannonian basin (Ujvári et al. 2012). This finding suggests that these samples probably contain some loess components considered as natural soil inputs, which is characteristic of the Pannonian basin (Ujvári et al. 2012). However, within this range, a particular sample (STN09: park), located close to the major north–south main road and relatively far from the CFPP (approx. 4 km) (Fig. 1b), shows a distinctly low ^87^Sr/^86^Sr ratio (0.713844) (Table 1). This low Sr isotopic ratio rather suggests additional anthropogenic source, such as smelter slag from the early ironworks, which could also be a component of multi-source pollution in Salgótarján (the ^87^Sr/^86^Sr isotopic ratio of smelter slag was not measured due to high disturbance and alteration across the city, but the material is clearly observed in form of reddish oxidized layers in this sample site; Fig. S6).

#### Evidence of additional sources on certain samples

A distinct group of samples fall below the loess range (Fig. 4, Table 1). These samples can be characterized by the lowest ^87^Sr/^86^Sr ratios including STN16 (playground), STN12 (kindergarten), and STN32 (roadside) with 0.710303, 0.711521, and 0.711573, respectively, despite not being the closest sampling sites to the CFPP (Fig. 4, Table 1). This can be explained by a significant influence from additional known anthropogenic sources (i.e., smelter slag), which is predominant in STN16 (playground) (Fig. S3), and incorporates Sr from carbonate and phosphate minerals with inherently low ^87^Sr/^86^Sr ratios, as described by Piatak et al. (2015), as well as contributions from additional unknown sources with even lower ^87^Sr/^86^Sr isotopic ratios than coal ash, likely transported by humans during land cover changes, which together offset the influence of coal ash in these samples listed above.

In contrast, the other group of samples, exceeding the loess range (Fig. 4) comprising STN22 (roadside), STN11 (park), and STN31 (roadside) exhibit the highest ^87^Sr/^86^Sr ratios of 0.718107, 0.718300, and 0.719559, respectively (Table 1). These samples are not farthest from CFPP, at distances of 1.8, 1.7, and 2.1 km (Fig. 4). The elevated ^87^Sr/^86^Sr in these samples can be attributed to urbanization, landscape change, and land-use change incorporating local soils with large amount of artificial infills, often composed of quartz-rich soils (Fig. S2). As explained earlier, such materials are commonly used for road construction and soil stabilization that are known to exhibit high ^87^Sr/^86^Sr ratios due to the high Rb content in their parent rocks (like granitic and metamorphic rocks), where radiogenic ^87^Sr is produced through the decay of ^87^Rb over geological timescales (e.g., Koeberl et al. 1998; Steinmann and Stille 1997).

### Semi-quantitative estimation of coal ash and local soil contributions to variations in ^87^Sr/^86^Sr across Salgótarján urban soils

**Fig. 5:**
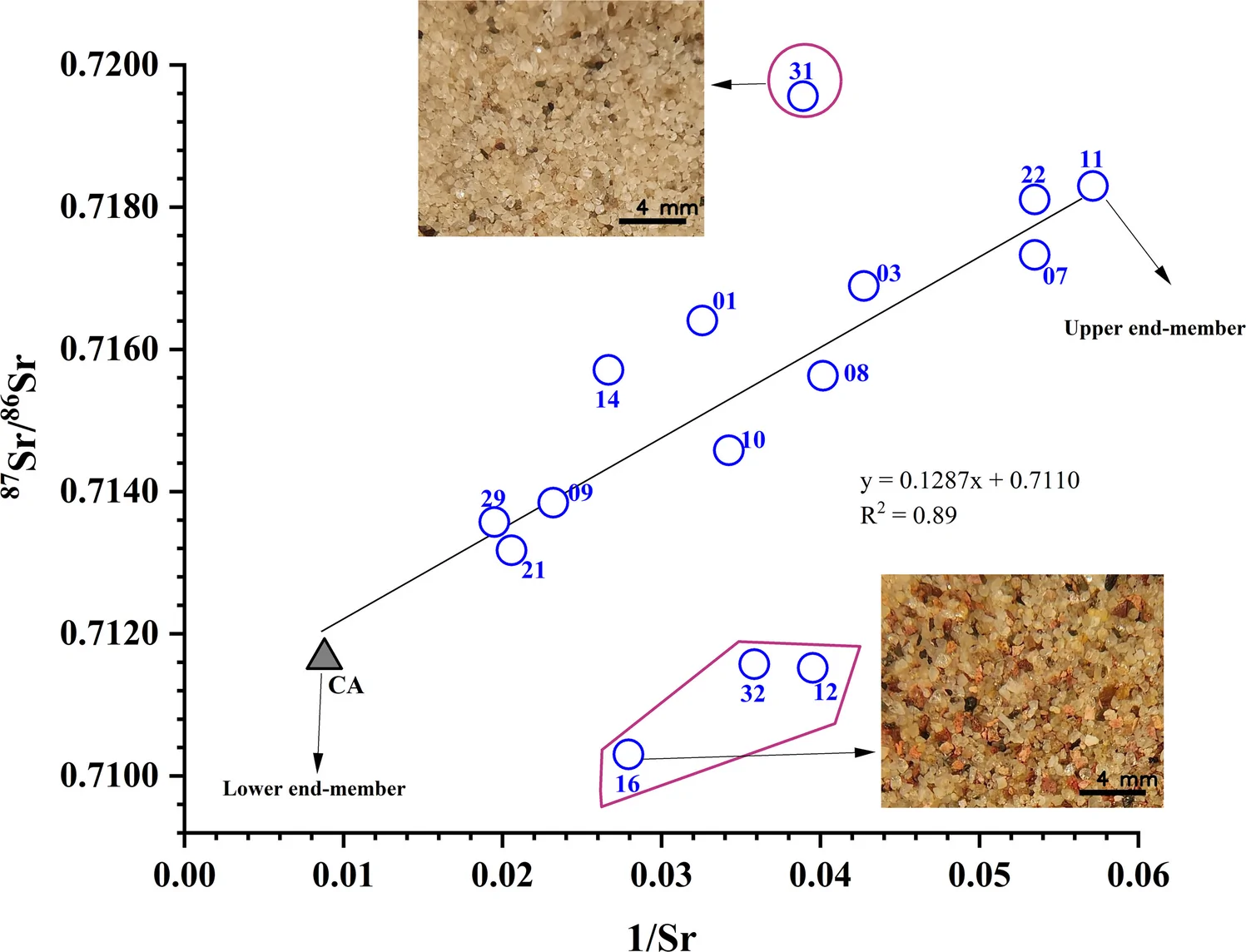
Variation of ^87^Sr/^86^Sr ratios vs. 1/Sr across diverse geographical locations and sample types. Blue open circles represent data points from Salgótarján urban soils. The stereo microscope images of two distinct samples: STN31 (roadside), dominated by quartz-rich grains (local soil), and STN16 (playground), characterized by coal ash and/or smelter slag content, highlighting compositional differences in the studied urban soils

The heterogeneity in ^87^Sr/^86^Sr ratios across Salgótarján urban soils, as discussed in the previous chapter, highlights the need to quantify the relative contributions of local soil and coal ash. To estimate the proportion of each source, the relationship between ^87^Sr/^86^Sr vs. 1/Sr (Fig. 5), clearly identifies two major end-members contributing to the observed ^87^Sr/^86^Sr ratios in Salgótarján urban soils. The coal ash (lower end-member), indicated with lower ^87^Sr/^86^Sr, whereas park urban soil/STN11/(upper end-member) shows a local soil consisting of natural or artificially made soil elevated in ^87^Sr/^86^Sr, which allows calculation of the semi-quantitative mixing ratio of two major potential sources (e.g., Widory et al. 2010). The significant variations in Salgótarján urban soil ^87^Sr/^86^Sr ratios reflect a complex mixing of local soil (e.g., natural soil/artificial infills), coal ash/smelter slag sources (Fig. 5, Table 1). Therefore, the samples are grouped into two categories based on their distinct isotopic characteristics: 1) those showing a strong positive correlation between ^87^Sr/^86^Sr vs. 1/Sr, indicating a clear mixing of two major sources as described by Wang et al. (2021), and 2) those which deviate from this correlation with the highest and lowest ^87^Sr/^86^Sr ratios compared to the two major end-members. Each group provides critical insights into the spatial distribution and relative contributions of these sources to the Salgótarján urban soils explained below.

#### Group 1: samples following strong linear correlation in ^87^Sr/^86^Sr vs. 1/Sr

In this study, a strong positive correlation (*R*^2^ = 0.89, *p* < 0.05) is observed for approximately 80% of Salgótarján urban soils of the NE part of the city (Fig. 5), suggesting that Sr variations are primarily driven by a mixing of two major end-members (i.e., local soil and coal ash). The lower end-member (coal ash—^87^Sr/^86^Sr: 0.711652) dominates in samples collected rather closer to the CFPP, contributes significantly to the lower ^87^Sr/^86^Sr observed in these samples (Figs. 4 and 5) as discussed above. In contrast, samples showing ^87^Sr/^86^Sr signatures closer to the upper end-member (STN11: park—^87^Sr/^86^Sr: 0.718300), suggest greater contribution from local soil (natural soil/artificial infills), or in other words, they show minimal or negligible influence from coal ash (Fig. 5).

The semi-quantitative contribution rates (%) of both coal ash and local soil, calculated using the two-end-member model (Table 2) (e.g., Lin et al. 2016; Zhao et al. 2019). The results indicate that urban soil samples, such as STN21 (roadside), STN29 (park), and STN09 (park) are primarily influenced by inputs from coal ash, with estimated contributions of 77%, 71%, and 67%, respectively. This contamination can be attributed to two pathways: first, the transport of coal ash through environmental geochemical processes. Since coal ash samples contain a mixture of fine and coarse particles (Table 1, Fig. 3), the fine particles (i.e., fly ash) are likely to be transported by wind and deposited through atmospheric deposition to nearby areas (e.g., Cohen et al. 2010). Furthermore, coal ash may also be introduced through its use as a filling material, sometimes along with smelter slag, during landscape changes as previously discussed for STN09 (park) (Fig. S6). Similarly, samples such as STN21 (roadside), STN29 (park), and STN14 (playground), which are located near former industrial sites (i.e., iron and steel works) (Fig. 1b), likely contain some influence from smelter slag in addition to coal ash (since Sr-isotopic ratio of smelter slag has not been directly measured, its contribution cannot be separately quantified in this model). In contrast, samples STN22 (roadside), STN07 (kindergarten), STN03 (kindergarten), and STN01 (kindergarten) show significant estimated contribution of local soil with 97%, 85%, 79%, and 71%, respectively (Table 2, Fig. S7).

**Table 2:** Semi-quantitative estimation of contribution rates (%) of coal ash and local soil in the Salgótarján urban soil samples calculated from the two-end-member model

Samples	Categories	Coal ash (%)	Local soil (%)
STN 01US	Kindergarten	29	**71**
STN 03US	Kindergarten	21	**79**
STN 07US	Kindergarten	15	**85**
STN 08US	Playground	40	60
STN 09US	Park	**67**	33
STN 10US	Playground	56	44
STN 14US	Playground	39	61
STN 21US	Roadside	**77**	23
STN 22US	Roadside	3	**97**
STN 29US	Park	**71**	29

#### Group 2: Samples with distinct ^87^Sr/^86^Sr ratios

Despite the prevalence of coal ash in studied STN urban soil samples, additional known anthropogenic sources, primarily smelter slag deposits, and unknown technogenic soils (human-transformed soils), contribute significantly to the heterogeneity in ^87^Sr/^86^Sr ratio of specific group of samples (STN12: kindergarten, STN16: playground, STN32: roadside) (Fig. 1b). This is observed by their significantly low ^87^Sr/^86^Sr ratios (Table 1, Fig. 5), which is particularly evident in STN16 (playground) with significantly lower ^87^Sr/^86^Sr ratio of 0.710303 than that of coal ash (0.711652) (Table 1, Fig. 5). This site, located in a former recreational area, contains multiple soil layers with varying compositions, showing oxidized grains including slag and coal ash (Fig. S3), suggesting old deposition of industrial by-products. Similarly, STN12 (kindergarten), situated at a site historically modified from a cottage into a kindergarten, contains transported materials for reconstruction, indicating mixed contamination rather than direct deposition of industrial waste. Moreover, STN32 (roadside), located near an area with a long history of industrial activity (Fig. 1b), features complex layers of slag deposits (Fig. S6), indicative of prolonged chemical alteration from past contamination. In contrast, STN31 (roadside) exhibits the highest ^87^Sr/^86^Sr ratio (0.719559), exceeding that of the upper end-member STN11 (0.718300) (Table 1, Fig. 5). This suggests the predominant presence of local soils, composed of high radiogenic ^87^Sr as described earlier, influenced by human disturbance during landscape changes. These findings highlight the influence of site-specific factors (mentioned above) on overall ^87^Sr/^86^Sr ratio of certain studied Salgótarján urban soils.

### Comparative studies worldwide

The ^87^Sr/^86^Sr ratios in Salgótarján urban soils are compared with findings from other countries (Fig. S8, Table 3) including not only urban soils but also surface sediments, as both reflect direct influence from anthropogenic contamination (e.g., Olivares et al. 2020), and studies on urban soils remain highly rare. For instance, comparing Salgótarján urban soil ^87^Sr/^86^Sr ratio results with those of urban soils from Bishkek and Teploklyuchenka cities (Kyrgyzstan) (Dewan et al. 2015), where major pollution sources include coal burning for residential heating and coal-based power stations (Dzushupov et al. 2022), we found statistically equivalent mean values (this study 0.715100 ± 0.002750, Kyrgyzstan 0.71577 ± 0.00023) (Table 1, Fig. S8). Notably, most urban soils in Kyrgyzstan, with higher ^87^Sr/^86^Sr ratios, likely influenced by nearby loess deposits (Dewan et al. 2015). Moreover, reported data from surface sediments collected from rivers flowing through Lianyungang City (China) and discharging into Haizhou Bay (Deng et al. 2021) also show a very similar mean value of ^87^Sr/^86^Sr ratio to that of Salgótarján urban soils, indicating that the area is affected by similar potential anthropogenic sources (e.g., coal combustion) to our studied area. This suggests a mixture of various anthropogenic sources with different portions which contributes to the mean value of ^87^Sr/^86^Sr ratios in the urban soils. The higher ^87^Sr/^86^Sr ratio observed in most of the surface sediments of the Jiulong River (China) (Lin et al. 2016), compared to present study, indicates that Sr accumulated in the surface sediments is predominantly from granite as natural (geogenic) source, exceeding the influence of anthropogenic source (e.g., coal combustion). The investigated urban soil samples derived from industrial and residential areas of Quanzhou (China) (Sun et al. 2018) indicate similar ^87^Sr/^86^Sr mean value to our results (Table 1, Fig. S8). This similarity highlights the significant influence of both anthropogenic activities and local soil inputs on forming the overall isotopic signature of the studied urban soils.

**Table 3:** Summary of minimum (min), mean ± standard deviation (SD) and maximum (max) values of ^87^Sr/^86^Sr ratios in urban soil (this study, Kyrgyzstan, China) and surface sediment samples (China)

Location	Type of samples	^87^Sr/^86^Sr			References
		min	mean ± SD	max	
This study	Urban soils (*n* = 15)	0.710303	0.715100 ± 0.002750	0.719559	This study
Bishkek and Teploklyuchenka (Kyrgyzstan)	Urban soils (*n* = 8)	0.71561	0.71577 ± 0.00023	0.71601	Dewan et al. (2015)
Jiulong (China)	Surface sediments (*n* = 14)	0.7164	0.7189 ± 0.0014	0.7209	Lin et al. (2016)
Quanzhou (China)	Urban soils (*n* = 4)	0.712380	0.714468	0.716851	Sun et al. (2018)
Haizhou Bay (China)	Surface sediments (*n* = 8)	0.7142	0.7154 ± 0.0008	0.7165	Deng et al. (2021)

## Conclusion remarks

This study examined Sr concentrations, physicochemical properties, and ^87^Sr/^86^Sr ratios in urban soils of Salgótarján (Hungary) to assess the potential influence of historical heavy industrial activities on soil geochemistry and environmental contamination. The findings from this study highlight the significant anthropogenic impact on urban soils in Salgótarján, with both coal ash and smelter slag occurring as dominant sources of contamination. These results underscore the role of industrial activities and urbanization on the chemical composition and isotopic signature of urban soils. The urban contamination not only affects soil composition but may also pose risks to local ecosystems and human health, especially in areas with higher exposure, such as playgrounds, parks, and kindergartens. The key conclusions from this study are summarized below:

1. The Sr concentrations (range 17.5–51.3 mg kg⁻^1^) in the urban soils suggest that proximity to emission sources (i.e., coal-fired power plant, iron and steel works) is linked with increased Sr accumulation.
2. Soil physicochemical properties, like TOC and TN contents, as well as pH values, and grain size distribution, can influence Sr concentration in the studied urban soils. Elevated TOC and TN levels in parks compared to those in kindergartens and playgrounds are attributed to greater vegetation coverage, increasing organic matter accumulation and, thus, enhancing Sr stabilization and concentration.
3. The enrichment in P and S content of certain urban soil samples is mainly associated with increased industrial by-products inputs, such as coal ash and smelter slag, as well as application of NPK fertilizers or the accumulation of urban organic waste, and dry/wet deposition of SO_2_ over long-term industrial activities.
4. A strong positive correlation (*R*^2^ = 0.86, *p* < 0.05) is observed between ^87^Sr/^86^Sr ratios and distance from the coal-fired power plant in over 50% of samples. This indicates that urban soil samples close to the coal-fired power plant show reduced ^87^Sr/^86^Sr ratios, reflecting high coal ash contribution. In contrast, with gradually increasing distance from the coal-fired power plant, higher ratios suggest gradually decreasing coal ash influence and a greater input from local soil (natural or artificially made soils).
5. In approximately 80% of studied urban soil samples, the semi-quantitative estimation from two-end-member model (^87^Sr/^86^Sr vs. 1/Sr; *R*^2^ = 0.89, *p* < 0.05) confirmed that coal ash serves as the dominant industrial input contributing its low ^87^Sr/^86^Sr footprint and high Sr concentration to the studied Salgótarján urban soils. In contrast, local soils (natural/artificially made soils), which are characterized by higher ^87^Sr/^86^Sr ratio and lower Sr concentration than coal ash, due to its high radiogenic ^87^Sr inputs (e.g., quartz-rich infill materials), contribute in varying portions to the studied urban soils, causing their ^87^Sr/^86^Sr ratios to exceed that of coal ash.

Overall, this study demonstrates the value of combining Sr concentrations with ^87^Sr/^86^Sr isotope ratios as a robust tracer for distinguishing natural and anthropogenic Sr sources in complex urban environments. The isotopic approach enables the identification of legacy industrial contamination associated with historical heavy industrial activities (i.e., coal-fired power plant), and variable contributions from local soil materials and other urban inputs. These findings illustrate the broader applicability of Sr isotope geochemistry as a tool for studying the long‑term environmental footprint of historical industrial activities in post‑industrial cities. This approach can support future urban soil monitoring programs, source apportionment studies, and urban land‑management strategies aimed at mitigating contamination risks and improving urban environmental quality, particularly in areas of elevated human exposure, such as playgrounds, parks, and kindergartens.

## Supplementary Information

Below is the link to the electronic supplementary material.

ESM 1 (DOCX 20.3 MB)

## Data Availability

All data generated or analyzed in this study are available within this article and its supplementary information files.

## Footnotes

1 Neogene brown coal is commonly referred to as “lignite” in literature corresponds to its classification as a low-rank coal characterized by relatively high moisture content and low heating value.

2 The Sr data for Ózd brown coal is obtained from the unpublished research database of the Ózd project at Lithosphere Fluid Research Lab (LRG), Institute of Geography and Earth Sciences, Eötvös Loránd University.

3 Artificially made soils are man-made substrates introduced during land use changes to enhance soil stabilization and compactness, commonly used in urban development (e.g., Bielská et al. 2012).
