# Supplementary material for: Strontium isotopic signatures and source apportionment in urban soils of Salgótarján: unraveling the impact of industrial emissions
Source: Environ Sci Pollut Res Int. 2026 Sep 23;33(30):15829–48. doi: 10.1007/s11356-026-38225-6 (PMC13633362; doi:10.1007/s11356-026-38225-6)
Supplement: Supplementary file 1 [file 11356_2026_38225_MOESM1_ESM.docx]

**Strontium isotopic signatures and source apportionment in urban soils of Salgótarján: unraveling the impact of industrial emissions**

**Mona Maghsoudlou^1,2^, Davaakhuu Tserendorj^1,3^, Gorkhmaz Abbaszade^1,4^,** **Norbert Kavasi^5,6^, Péter Völgyesi^3^, Edit Tóth-Bodrogi^7^, Sarata K. Sahoo^8,9^, Marko Štrok^6^, Kazumasa Inoue^8^, Tibor Kovács^7^, Csaba Szabó^1,10,11^**

^1^Lithosphere Fluid Research Lab, Institute of Geography and Geology, Eötvös Loránd University, Pázmány Péter sétány 1/C, Budapest, 1117, Hungary

^2^HUN-REN, Geographical Institute, Research Centre for Astronomy and Earth Sciences, Budaőrsi út 45, Budapest H-1112, Hungary

^3^HUN-REN, Centre for Energy Research, Nuclear Security Department, Konkoly Thege út 29-33, Budapest, 1121, Hungary

^4^Flow Cytometry Group, Department of Applied Microbial Ecology, Helmholtz Centre for Environmental Research GmbH – UFZ, Permoserstraße 15, 04318 Leipzig, Germany

^5^Regional Environmental Co-creation Unit, Fukushima Institute for Research, Education and Innovation, Fukushima, 960-1295, Japan

^6^Department of Environmental Sciences, Jožef Stefan Institute, Ljubljana 1000, Slovenia

^7^Institute of Radiochemistry and Radioecology, University of Pannonia, Veszprém, Hungary

^8^Tokyo Metropolitan University, 7-2-10 Higashiogu, Arakawa-ku, Tokyo 116-8551, Japan

^9^National Institutes for Quantum Sciences and Technology (QST), 4-9-1 Anagawa, Inage-ku, Chiba 263-8555, Japan

^10^HUN-REN, Institute of Earth Physics and Space Science, Sopron, Hungary

^11^Centre of Environmental Sciences, Eötvös Loránd University, Pázmány Péter sétány 1/A, 1117, Budapest, Hungary

Corresponding Author: Péter Völgyesi

[volgyesi.peter@ek.hun-ren.hu](mailto:volgyesi.peter@ek.hun-ren.hu)

Phone: 0036-1-3922222 ext. 2604

Mobile: (+36) 20-311-3299

**Fig. S1** Spatial distribution of Sr concentration (mg kg ^-1^) in the northeastern part of Salgótarján. The radii of the circles are proportional to the Sr concentrations (mg kg ^-1^) at each sampling site. Location of former industrial sites, slag dumps, including smelter slag hill (S.S.) and coal ash heaps (C.A.), as well as sampling point of coal ash, brown coal and coal-fired power plant are also shown

STN32

10 cm

**STN31 (Roadside)**

Debris of bricks

Stereo microscope image

**Fig. S2** Stereo microscope image of STN31 (roadside) soil sample sieved to <2 mm, showing the predominant of artificial soil infills during landscape changes

**STN16 (playground)**

Coal ash/smelter slag deposits

Stereo microscope image

**Fig. S3** Stereo microscope image of STN16 (playground) soil sample sieved to <2 mm, showing the predominant of coal ash/smelter slag contents

STN29

STN11

**Fig. S4** Examples of surrounding environments of some park samples; STN29 (left) and STN11 (right), indicating the influence of land use and land cover changes, with dense vegetation cover that incorporates high organic matter in these samples

**Fig. S5** Grain size distribution of studied Salgótarján urban soils

STN09

10 cm

10 cm

STN32

**Fig. S6** Representative examples of contaminated urban soils. Only the upper 10 cm of soil was collected for analysis after removing surface vegetation cover. (Left: STN09 – park): well-mixed, high coal ash/oxidized smelter slag content. (Right: STN32 – roadside): complex layering, indicating multiple accumulation phases of coal ash/smelter slag

10 cm

STN07

10 cm

STN11

10 cm

STN22

STN11

**Fig. S7** Representative examples of uncontaminated urban soils including (left: STN11 – Park), STN07 (middle: STN07 – Kindergarten), and (right: STN22 – Roadside) representative of local soil with no visible coal ash or smelter slag influence

**Fig. S8** Variation of ^87^Sr/^86^Sr ratios vs. Sr concentrations across diverse geographical locations and sample types. Blue open circles represent data points from Salgótarján urban soils, red closed circles indicate Quanzho urban soils (Sun et al., 2018), blue closed triangles depict samples from Kyrgyzstan urban soils (Dewan et al., 2015), green closed squares represent Jiuling river surface sediments (Lin et al., 2016), and purple rhombi depict data from Haizhou bay surface sediments (Deng et al., 2021)

**Table S1** Analytical quality control of **⁸**⁷Sr/⁸⁶Sr measurements using the certified reference material (CRM) NIST-SRM-987

| **Sample ID** | **Category** | **^87^Sr/^86^Sr** | **Internal precision (within-run repeatability)** | | | **Measurement basis** |
| --- | --- | --- | --- | --- | --- | --- |
|  |  |  | **Std Err% ^a^** | | **2SE ^b^** |  |
| STN 01US | Kindergarten | 0.716405 | 0.000213 | | 0.000003 | Measurement cycles within each analytical run |
| STN 03US | Kindergarten | 0.716890 | 0.000231 | | 0.000003 | Measurement cycles within each analytical run |
| STN 07US | Kindergarten | 0.717330 | 0.000202 | | 0.000003 | Measurement cycles within each analytical run |
| STN 12US | Kindergarten | 0.711521 | 0.000200 | | 0.000003 | Measurement cycles within each analytical run |
| STN 08US | Playground | 0.715630 | 0.000213 | | 0.000003 | Measurement cycles within each analytical run |
| STN 10US | Playground | 0.714581 | 0.000377 | | 0.000005 | Measurement cycles within each analytical run |
| STN 14US | Playground | 0.715711 | 0.000293 | | 0.000004 | Measurement cycles within each analytical run |
| STN 16US | Playground | 0.710303 | 0.000312 | | 0.000004 | Measurement cycles within each analytical run |
| STN 09US | Park | 0.713844 | 0.000171 | | 0.000002 | Measurement cycles within each analytical run |
| STN 11US | Park | 0.718300 | 0.000310 | | 0.000004 | Measurement cycles within each analytical run |
| STN 29US | Park | 0.713573 | 0.000249 | | 0.000004 | Measurement cycles within each analytical run |
| STN 21US | Roadside | 0.713175 | 0.000206 | | 0.000003 | Measurement cycles within each analytical run |
| STN 22US | Roadside | 0.718107 | 0.000293 | | 0.000004 | Measurement cycles within each analytical run |
| STN 31US | Roadside | 0.719559 | 0.000193 | | 0.000003 | Measurement cycles within each analytical run |
| STN 32US | Roadside | 0.711573 | 0.000277 | | 0.000004 | Measurement cycles within each analytical run |
| STN CA | Coal ash | 0.711652 | 0.000183 | | 0.000003 | Measurement cycles within each analytical run |
| STN BC | Brown coal | 0.712830 | 0.000231 | | 0.000003 | Measurement cycles within each analytical run |
| NIST-SRM-987 (run1) | Standard | 0.710253 | 0.000266 | | 0.000004 | Measurement cycles within each analytical run |
| NIST-SRM-987 (run 2) | Standard | 0.710255 | 0.000300 | | 0.000004 | Measurement cycles within each analytical run |
| NIST-SRM-987 | Certified value | 0.71034 ± 0.00026^e^ |  | |  |  |
| NIST-SRM-987 | Recommended value | 0.710248± 0.000012 **^f^** |  | | |  |
| **Sample ID** | **Category** | **^87^Sr/^86^Sr** | **External precision (long-term analytical reproducibility)** | | | **Measurement basis** |
|  |  |  | **Std Dev% ^c^** | **2SD ^d^** | |  |
| NIST-SRM-987 (long-term run) | Standard | 0.710249 | 0.000939 | 0.000013 | | 280 independent loads and  41,505 measurement cycles for 13 years |

**^a^** **Std Err%:** standard error of the mean (%), characterizing the uncertainty associated with the estimated mean from repeated measurement cycles within each analytical run (Taylor & Kuyatt, 1994)

**^b^** **2SE:** two standard errors of the mean, representing internal precision under repeatability conditions, calculated from repeated measurement cycles within each analytical run (Taylor & Kuyatt, 1994; ISO 5725-1:2023)

**^c^** **Std Dev%:** standard deviation relative to the mean (%) (Taylor & Kuyatt, 1994), characterizing the scatter of repeated long-term measurements of NIST-SRM-987 over the 13-year monitoring period

**^d^** **2SD:** two standard deviations, representing the external precision (long‑term analytical reproducibility) (ISO 5725‑1:2023), calculated from repeated long-term measurements of NIST-SRM‑987 over the 13‑year monitoring period

**^e^** **Certified value:** NIST-SRM-987 certified ⁸⁷Sr/⁸⁶Sr value of 0.71034 ± 0.00026 (expanded uncertainty, k=2) (NIST, 2007)

**^f^** **Recommended value:** Consensus ^87^Sr/^86^Sr reference value of 0.710248 ± 0.000012 for NIST-SRM‑987 (Deines et al., 2003; McArthur et al., 2025)

**Table S2** Analytical quality control of elemental concentration measurements using the certified reference material (CRM) STD DS11

| **STD DS11** | **Sr (mg/kg)** | **P (m/m%)** | **S (m/m%)** |
| --- | --- | --- | --- |
| Measured values (n = 3) | 69.0 / 71.7 / 64.0 | 0.075 / 0.072 / 0.069 | 0.28 / 0.27 / 0.26 |
| Mean ± SD | 68.2 ± 3.9 | 0.0720 ± 0.0030 | 0.270 ± 0.010 |
| Certified value | 67.3 | 0.0701 | 0.2835 |
| Relative bias (RB %)* | 1.34 % | 2.71 % | 4.76 % |
| Relative standard deviation (RSD %)** | 5.72 % | 4.17 % | 3.70 % |

*Relative bias (RB %) = (|Mean − Certified| / Certified) × 100, expressing trueness (closeness to the accepted reference value) as defined in ISO 5725-1:1994

**Relative standard deviation (RSD %) = (SD / Mean) × 100, expressing precision (scatter of replicate measurements) as defined in ISO 5725-1:1994

| Location | Brown coal formation | Sr concentration (mg kg⁻¹) | | References |
| --- | --- | --- | --- | --- |
|  |  | Range | value/mean |  |
| Salgótarján (Hungary) | Neogene brown coal (n=1) | - | 52.8 | This study |
| Ózd (Hungary) | Neogene brown coal (n=1) | - | 66.1 | This study |
| Nováky, Handlová (Slovakia) | Neogene brown coal (n=8) | 55.1 - 71.4 | 62.6 | O’Keefe et al., (2011) |
| Marica-East, Oranovo, Kanina (Bulgaria) | Late Miocene to Eocene brown coal (n=86) | 82 - 132 | 95 | Eskenazy & Minčeva (1989) |
| Kovin (Serbia) | Upper Miocene brown coal (n=44) | 46.04 - 253.06 (seam I) | 125 | Životić et al., (2019) |
|  |  | 10.19 - 176.12 (seam II) |  |  |
|  |  | 121 - 219 (seam III) |  |  |
| Rio Maior Basin (Portugal) | Pliocene brown coal (n=4) | 34.5 - 76.0 | 47 | Suárez-Ruiz et al., (2006) |

**Table S3** Summary of strontium concentration (mg kg⁻¹) (range, mean) in brown coal samples, accross different Central-Eastern Europe regions, including this study, Salgótarján and Ózd (Hungary), and comparative data from Nováky, Handlová (Slovakia) (O’Keefe et al., 2011) , MariSa-East, Oranovo, Kanina (Bulgaria) (Eskenazy & Minčeva, 1989), Kovin (Serbia) (Životić et al., 2019), as well as Southern Europe data from Rio Maior Basin (Portugal) (Suárez-Ruiz et al., 2006) are shown

| Location | Coal Type | Sr concentration (mg kg⁻¹) | | References |
| --- | --- | --- | --- | --- |
|  |  | Range | Mean ± SD (if any) |  |
| Salgótarján (Hungary) | Brown coal | 17.5 - 51.3 | 30.6 ± 10.3 | This study |
| Bitola (Macedonia) | Brown coal | 3.6 - 420 | 89 | Stafilov et al., (2018) |
| West Bengal (India) | Brown coal | - | 56.8 ± 4.3 | George et al., (2015) |
| Savannah River (Georgia, USA) | Brown coal | Up to 60 | - | Evans et al., (1980) |
| Inner Mongolia (China) | Brown coal | 148 - 201 | 172 ± 17 | Gong et al., (2018) |
| Setúbal (Portugal) | Bituminous coal | 20 - 299 | 95 ± 91 | Dinis et al., (2021) |

**Table S4** Summary of Sr concentrations (mg kg⁻¹) (range, mean ± standard deviation (SD)) in urban soil samples around the world, including this study (Salgótarján, Hungary), and comparative data from Bitola (Macedonia) (Stafilov et al., 2018), West Bengal (India) (George et al., 2015), Savannah River (Georgia, USA) (Evans et al., 1980), Inner Mongolia (China) (Gong et al., 2018), and Setúbal (Portugal) (Dinis et al., 2021), highlighting the influence of coal ash on Sr concentration distributions in urban soils

**References**

Deines, P., Goldstein, S. L., Oelkers, E. H., Rudnick, R. L., & Walter, L. M. (2003). Standards for publication of isotope ratio and chemical data in Chemical Geology. *Chemical Geology*, *202*(1), 1–4. https://doi.org/https://doi.org/10.1016/j.chemgeo.2003.08.003

Dinis, M. de L., Fiúza, A., Góis, J., de Carvalho, J. S., & Meira Castro, A. C. (2021). Assessment of natural radioactivity, heavy metals and partic-ulate matter in air and soil around a coal-fired power plant—an integrated approach. *Atmosphere*, *12*(11). https://doi.org/10.3390/atmos12111433

Eskenazy, G., & Minčeva, E. (1989). On the geochemistry of strontium in Bulgarian coals. *Chemical Geology*, *74*(3–4), 265–276. https://doi.org/10.1016/0009-2541(89)90037-5

Evans, D. W., Wiener, J. G., & Horton, J. H. (1980). Trace Element Inputs from a Coal Burning Power Plant to Adjacent Terrestrial and Aquatic Environments. *Journal of the Air Pollution Control Association*, *30*(5), 567–573. https://doi.org/10.1080/00022470.1980.10465982

George, J., Masto, R. E., Ram, L. C., Das, T. B., Rout, T. K., & Mohan, M. (2015). Human Exposure Risks for Metals in Soil Near a Coal-Fired Power-Generating Plant. *Archives of Environmental Contamination and Toxicology*, *68*(3), 451–461. https://doi.org/10.1007/s00244-014-0111-x

Gong, B., Yong, Q., Xiong, Z., Tian, C., Yang, J., Zhao, Y., & Zhang, J. (2018). Mineral matter and trace elements in ashes from a high-arsenic lignite fired power plant in Inner Mongolia, China. *International Journal of Coal Geology*, *196*(August), 317–334. https://doi.org/10.1016/j.coal.2018.08.003

International Organization for Standardization. (2023). Accuracy (trueness and precision) of measurement methods and results — Part 1: General principles and definitions (ISO Standard No. 5725-1:2023). https://www.iso.org/standard/69418.html

McArthur, J. M., Millar, I. L., Drury, A. J., & Wagner, D. (2025). Strontium-isotope stratigraphy: methodology, standard values (SRM987, EN-1, E&A), a new Neogene curve of 87Sr/86Sr against time, its implications for astrochronology ([I]ODP Sites 1146, 1264, U1337, and U1338), and its application to ODP Site 758 (Indian . *Palaeogeography, Palaeoclimatology, Palaeoecology*, *669*, 112907. https://doi.org/https://doi.org/10.1016/j.palaeo.2025.112907

National Institute of Standards and Technology. (2007). Certificate of analysis: Standard Reference Material® 987 strontium carbonate (isotopic standard). U.S. Department of Commerce. https://www.nist.gov/srm

O’Keefe, J. M. K., Hower, J. C., Finkelman, R. F., Drew, J. W., & Stucker, J. D. (2011). Petrographic, geochemical, and mycological aspects of Miocene coals from the Nováky and Handlová mining districts, Slovakia. *International Journal of Coal Geology*, *87*(3–4), 268–281. https://doi.org/10.1016/j.coal.2011.06.019

Stafilov, T., Šajn, R., Arapčeska, M., Kungulovski, I., & Alijagić, J. (2018). Geochemical properties of topsoil around the coal mine and thermoelectric power plant. *Journal of Environmental Science and Health - Part A Toxic/Hazardous Substances and Environmental Engineering*, *53*(9), 793–808. https://doi.org/10.1080/10934529.2018.1445076

Suárez-Ruiz, I., Flores, D., Marques, M. M., Martinez-Tarazona, M. R., Pis, J., & Rubiera, F. (2006). Geochemistry, mineralogy and technological properties of coals from Rio Maior (Portugal) and Peñarroya (Spain) basins. *International Journal of Coal Geology*, *67*(3), 171–190. https://doi.org/10.1016/j.coal.2005.11.004

Taylor, B. N., & Kuyatt, C. E. (1994). NIST Technical Note 1297 1994 Edition, Guidelines for Evaluating and Expressing the Uncertainty of NIST Measurement Results. *National Institute of Standards and Technology*, 1–20.

Životić, D., Cvetković, O., Vulić, P., Gržetić, I., Simić, V., Ilijević, K., Dojčinović, B., Erić, S., Radić, B., Stojadinović, S., & Trifunović, S. (2019). Distribution of major and trace elements in the Kovin lignite (Serbia). *Geologia Croatica*, *72*(1), 51–79. https://doi.org/10.4154/gc.2019.06
